# National Military Training

**Published:** 1913-10-25

**Authors:** 


### NATIONAL MILITARY TRAINING.

Those who are still under the impression that the introduction of systematic physical exercise in schools, exercise of the kind and character associated with military drill and squad evolutions, is responsible, or would be responsible, for an orgy of militarism, would do well to read the latest report of the Minister of Public Instruction for Victoria. Helped by instructors from the Defence Department, who have organised special courses for teachers, the schools in the province are now able to institute courses of drill which are in every way satisfactory. We have frequently referred to the absurdity of objectless physical exercises. Our own Board of Education has issued an elaborate manual which instructs the school teacher how to set about teaching and supervising physical exercises for boys and girls. This syllabus is carefully, sometimes meticulously, followed by teachers in all the schools under the Board. Yet with what result? One has only to read the reports of the various medical officers of schools to find that physical exercises, as given in our schools, leave much, very much, to be desired. The main reason for this is because such exercise is objectless. The child knows perfectly well that it is routine exercise; it is in the nature of a lesson, which, unless the teacher is exceptionally individualistic, degenerates into dull, mechanical movements, aimless except so far as the immediate execution of a particular evolution is concerned. Above all, it fails to create that spirit of solidarity, of communal interdependence, which is the aim and object of so-called ceremonial drill, which, again, is merely military exercises under another name. Teachers feel this want in the present system, and openly express their preference for even such modified military training as they were allowed to give under the old School Boards. But against them stands the iron prejudice that sees in such training the thin end of the wedge that will open the door to that miserable bogey of militarism. In Victoria they think differently. "Already the good effect of the drill," writes the Minister, "is noticeable in many schools, in the better carriage of the scholars, and in their improved bodily positions adopted during the ordinary school lessons. . . . Means will be taken next year, by examination and measurement of scholars, to ascertain the developmental effect of the new physical training. The training of the junior cadets is being carried on in accordance with the regulations of the Commonwealth Defence Department. It is satisfactory to note that the district inspectors and State school teachers have taken up the work for the Defence Department with commendable enthusiasm." The photographs illustrating the report, and the notes of the departmental inspectors attached, fully bear out the first statement.

Military drill and discipline are the outcome of years of experiment in which the defects of other systems have been discarded and the good points modified and improved. When we add to them such exercises and sports as rifle shooting, signalling, and ambulance training, we get a course of physical exercises which leaves little, if anything, to be desired so far as interesting objectives, inculcation of valuable lessons of patience and self-control, and harmonious co-operation between different units are concerned. In the self-governing Dominions the introduction of the Defence Acts, which make military training compulsory, has cut the Gordian knot and compelled the Boards of Education to re-cast their schemes. In Victoria, for example, this has been done with the best possible results by modifying our own Board of Education syllabus by the inclusion in the course of military drill. With us, since there seems no hope in the near future that we shall emulate the Dominions in making every school-boy sufficiently cognisant of his future duty as a citizen, at least the Board of Education might allow such teachers as desire to introduce modified military training into their schools. It is one thing to prohibit compulsory training of this sort; it is quite another to prevent voluntary effort from initiating a beneficial reform.

